# Increased Matrix Stiffness Promotes Slow Muscle Fibre Regeneration After Skeletal Muscle Injury

**DOI:** 10.1111/jcmm.70423

**Published:** 2025-02-19

**Authors:** Dongmei Wang, Jiahong Wu, Zeyu Xu, Jinning Jia, Yimei Lai, Zhihua He

**Affiliations:** ^1^ Department of Physical Education Anhui University of Technology Maanshan Anhui China; ^2^ Department of Medicine Sun Yat‐Sen University Shenzhen Guangdong China; ^3^ Department of Pathology The First Affiliated Hospital of Gannan Medical University Ganzhou Jiangxi China; ^4^ Institute of Urology The First Affiliated Hospital of Gannan Medical University Ganzhou Jiangxi China

**Keywords:** cytoskeleton, extracellular matrix, mitochondrial fission, muscle fibre type, RHO/ROCK pathway, stiffness

## Abstract

The global prevalence of skeletal muscle diseases has progressively escalated in recent years. This study aimed to explore the potential role of matrix stiffness in the repair mechanisms following skeletal muscle injury. We observed an increase in muscle stiffness, a significant rise in the number of type I muscle fibres and a notable elevation in mRNA expression levels of *Myh7/2* alongside a decrease in *Myh1/4* on day 3 post tibialis anterior muscle injury. To replicate these in vivo changes, C2C12 cells were cultured under high matrix stiffness conditions, and compared to those on low matrix stiffness, the C2C12 cells cultured on high matrix stiffness showed increased expression levels of *Myh7*/*2* mRNA and production levels of MYH7/2, indicating differentiation into slow‐twitch muscle fibre types. Furthermore, up‐regulation of DRP1 phosphorylation along with elevated F‐actin fluorescence intensity and RHOA and ROCK1 production indicates that high matrix stiffness induces cytoskeletal remodelling to regulate mitochondrial fission processes. Our data also revealed up‐regulation in mRNA expression level for *Actb*, phosphorylation level for DRP1, mitochondrial quantity and MYH7/2 production level. Importantly, these effects were effectively reversed by the application of ROCK inhibitor Y‐27632, highlighting that targeting cytoskeletal dynamics can modulate myogenic differentiation pathways within C2C12 cells. These findings provide valuable insights into how matrix stiffness influences fibre type transformation during skeletal muscle injury repair while suggesting potential therapeutic targets for intervention.

**Abbreviations**
DRP1: Dynamin‐related protein 1
ECM: Extracellular matrix
MYHC: Myosin heavy chain
SCs: Satellite cells
TA: Tibialis anterior

## 1 Introduction

The motor system is composed of bones, joints and soft tissues. Soft tissues include skeletal muscles, tendons, ligaments, fascia, etc. Skeletal muscle is the most important part of soft tissue. It is composed of muscle fibres, extracellular matrix rich in laminin and collagen, different cell populations, blood vessels and nerves, etc. After injury, it responds to injury and undergoes a highly coordinated process of degeneration and regeneration at the tissue, cellular and molecular levels [1]. Studies have shown that the prevalence of musculoskeletal diseases in the world increased from 1990 to 2019 [2], and the prevalence was the highest among global diseases in 2019 [3]. Therefore, it is of great significance to deeply analyse the pathological process of skeletal muscle injury repair and explore effective clinical intervention methods.

Satellite cells (SCs) are a specific population of adult stem cells in skeletal muscle, which are located below the basement membrane of skeletal muscle fibres [4] and are usually in a quiescent state. Quiescent SCs express Pax7, silencing transcription factors involved in myogenic differentiation [5, 6], inducing SCs proliferation and self‐renewal, and reducing their differentiation potential [7, 8, 9]. When muscle tissue is severely damaged by trauma or genetic defects, SCs are activated and undergo asymmetric division to generate SCs and satellite‐directed progenitor cells, the former reconstitute the stem cell pool through self‐renewal, and the latter proliferate and differentiate to form myoblasts [1, 10]. Myoblasts are characterised by the production of myogenic regulatory factors (MyoD, Myf5, Myogenin and MRF4) [11, 12, 13, 14], which promote differentiation and fusion to form multinucleated myotubes, construct neuromuscular junctions and ultimately complete muscle regeneration.

As a highly dynamic structural network, the extracellular matrix (ECM) provides a microenvironment for tissue formation and repair [15]. The ECM is mainly composed of fibroforming proteins such as collagen, elastin, fibronectin and glycoprotein [16], which can be divided into two types according to their location and composition: interstitial matrix and basement membrane (special forms of ECM) [17]. The ECM of skeletal muscle contains three layers, and the innermost structure is the basement membrane [18], which wraps around and connects directly to muscle fibres and is involved in supporting and regenerating muscles [19, 20, 21]. Matrix stiffness, as a mechanical property of ECM, is determined by the content and density of cross‐linked proteins, including collagen, fibronectin and laminin [22]. Numerous literatures have demonstrated that matrix stiffness plays an important role in the migration [23, 24], proliferation [25] and differentiation [26, 27] of muscle stem cells.

Myosin heavy chain (MYHC) isoforms are the most commonly used classification criteria for muscle fibres. Human limb muscles contain three MYHC isoforms, namely type I (MYH7), type IIa (MYH2) and type IIx (MYH1), while the muscle of the mouse also contains type IIb (MYH4) [28, 29]. Type I fibre, also known as slow muscle fibre, mainly performs oxidative metabolism and is suitable for endurance exercise. Type IIb and IIx, also known as fast muscle fibres, are mainly glycolytic and suitable for short‐term strength exercise. Type IIa fibres are intermediate fibres with mixed metabolic patterns that can produce high power at a considerable speed while having good endurance [28, 30]. Due to the induction of high plasticity of muscle fibre types by mechanical loading [28]. Therefore, exploring whether matrix stiffness can promote muscle fibre type transformation after injury to repair muscle can be used as an important treatment for muscle injury repair.

## 2 Methods

### 2.1 Experimental Animal Grouping and Modelling

C57BL/6J male mice aged 6–8 weeks were purchased from Shanghai Model Organisms Center Inc. This study was approved by the Experimental Animal Management and Use Committee of Shenzhen TopBiotech Co. Ltd. (TOPGM‐IACUC‐2024‐0057). The mice were randomly assigned to Control group, 3 days after injury (3 DPI) group and treatment (3 DPI + Y) group, with five mice in each group. In 3 DPI + Y group, Y‐27632 (5 mg kg) was intraperitoneally injected at 15 min before modelling. The legs of mice in 3DPI group and 3DPI + Y group were cut, and the anterior tibial muscle (TA) was exposed. Then TA muscle was frostbitten with a cotton swab dipped in liquid nitrogen at −80°C, and the wound was sutured. The mice in the control group were treated with the same incision suture.

### 2.2 Tissue Collection and Sectioning

After successful modelling, the mice were sacrificed by cervical dislocation, and the TA muscles of the mice were harvested. TA muscles for Atpase staining were fixed in 4% paraformaldehyde, embedded in OCT and made into frozen sections. TA muscle for muscle mechanical properties detection and immunofluorescence staining were embedded with OCT and made into frozen sections. TA muscles used for Western blot experiments were placed in EP tubes and stored in an ultra‐low temperature freezer at −80°C.

### 2.3 Preparation of Different Stiffness Gel Matrices

According to the gel ratio formula of 10 kPa and 100 kPa: 40%w/v acrylamide, 2%w/v diacrylamide, 1 M HEPES and ddH_2_O were added, and the coagulant (10% ammonium persulfate and TEMED) was added, mixed and injected into the WB plate to stand. The gel was removed and immersed in HEPES, cut and paved well plates, cleaned with HEPES and PBS, added with Sulfo‐SANPH violet crosslinking agent, ultraviolet light, after cleaning, rat tail type I collagen was added, the gel was washed and added with sterile PBS, sterilised under ultraviolet light and waiting for cell inoculation.

### 2.4 Cell Culture and Experimental Groupings

The C2C12 myoblasts were cultured in DMEM medium (containing 10% fetal bovine serum +1% penicillin/streptomycin) and placed in a 37°C, 5% CO_2_ cell incubator. The DMEM medium was changed every 2 days. When the cell density reached 80%–90%, the cells were digested with 0.25%w/v trypsin for cell passage or differentiation experiments. The differentiation medium was DMEM medium (containing 2% horse serum +1% penicillin/streptomycin), and the differentiation medium was changed once a day. C2C12 cells were randomly divided into 10 kpa group and 100 kpa group, seeded on the hydrogel with 10 kpa and 100 kpa stiffness, respectively, and the differentiation medium was added, and subsequent experiments were performed after 3 days of culture. To inhibit ROCK kinase in vitro, the differentiation medium of the 100 kpa group was pretreated with 10 μM Y‐27632 (MedChemExpress, USA) for 1 day, depending on the final liquid volume of cultured cells.

### 2.5 Mechanical Characterisation of Muscle Tissue

AFM was performed on an Asylum Research Cypher AFM (Oxford Instruments, USA) to measure spatial variations in the mechanical properties of control and injured tissue samples. Tissue sections were immersed in PBS at 25°C using a cantilever scaffold and platform. Triangular SiN cantilevers with sharp Si tips were used both to minimise the interaction volume between measurements (nominal radius *R* ≈ 2 nm) and to use a tip with a near‐conical shape (nominal half‐angle *α* ≈ 22°). The spring constant kc of each cantilever was measured with the thermal fluctuation method (the final values of kc ranged from 0.112 to 0.148 N/m, uncertainty of ≈ 0.002 N/m). Young's modulus plots were generated by performing force spectral analysis at each point in a 64 × 16 grid over a 5 μm × 1.25 μm region (≈ 80 nm pixel size).

### 2.6 ATPase Staining

Muscle fibre types (type I and type II) were detected by GENMED Atpase Frozen Section Human Muscle tissue Typing Staining kit (GENMED SCIENTIFICS INC, USA): Sample activation treatment (successively added GENMED alkaline solution, GENMED reaction working solution, GENMED activation solution), sample staining treatment (successively added GENMED displacement solution, GENMED cleaning solution, GENMED chromogenic solution, GENMED cleaning solution), sealed slides and observed under a general light microscope. Among them, type I muscle fibres stained light brown and type IIa/IIb muscle fibres stained dark brown.

### 2.7 Real‐Time Fluorescence Quantitative PCR

Total cellular RNA was extracted using Trizol solution, and 1 μg of mRNA was reverse transcribed into cDNA using the HiFiScript gDNA RemovalRT MasterMix kit (CWBIO, CN). The PCR reaction solution (cDNA, primer and SYBR Green) was prepared and amplified on a PCR instrument. The reaction conditions were as follows: predenaturation at 95°C for 30 s; 40 cycles of denaturation at 95°C for 5 min, annealing at 60°C and extension for 37 s were performed. The CT value of fluorescence quantification was read by software, and the relative quantitative analysis was performed: the relative expression of target gene = $2^{-{\mathrm{\Delta \Delta C}}_{T}}$. Gene transcript levels were normalised to the expression level of the Gapdh transcript. The primers used were as follows: *Myh7* (Forward: CTCCAGGGGTGATGGACAAC; Reverse: CGATACCTCTGCCGGAAGTC), *Myh2* (Forward: GTGGTGGAGCTGCCAAGAAA; Reverse: ACGAAATGAGGATGGGTGCT), *Myh1* (Forward: CTCCTCCACACCCAGAACAC; Reverse: TGTCCTCCATCTCTCCCTGG), *Myh4* (Forward: TTTAAAGCCGGCCTGTTGGG; Reverse: GCGGACGTTGTACTGAATGC), *MT‐CO2* (Forward: CAAACCTACGCCAAAATCCA; Reverse: GAAATGAATGAGCCTACAGA), *Actb* (Forward: GATATCGCTGCGCTGGTCG; Reverse: CATTCCCACCATCACACCCT), *Gapdh* (Forward: GGAGAGTGTTTCCTCGTCCC; Reverse: ATGAAGGGGTCGTTGATGGC).

### 2.8 Western Blot

Protein was extracted from the TA muscle tissue and C2C12 cells using RIPA lysis buffer containing 1% PMSF and 0.5% proteinase/phosphatase inhibitors. The protein concentration was determined using the BCA assay kit. The samples were then subjected to conventional SDS‐PAGE, electrophoresis, blocking and incubation with primary antibodies (MYH7/2/4/1, 1:1000; p‐DRP1, 1:1000; RHOA, 1:1000; ROCK1, 1:1000; internal standard GADPH, 1:1000) at 4°C overnight. The membranes were then washed with TBST, incubated with secondary antibodies (1:5000) at room temperature for 1 h. The membranes were then placed on a gel imaging apparatus and exposed to developing solution. The protein levels were analysed using Image J.

### 2.9 ATP Content Determination

ATP content determination uses ATP content detection kit (BOXBIO, CN): centrifuge cells, add extraction solution to the sample (cell number (104): extraction solution volume (mL) = 103:1), chill and sonicate in an ice bath, centrifuge at 4°C at 10,000 g for 10 min, transfer the supernatant, add chloroform, centrifuge at 4°C at 10,000 g for 5 min, transfer the supernatant to an ice bath and wait for measurement. In a 1 mL quartz cuvette, add the sample/standard dilution solution, reagent one and detection working solution in sequence. Determine the absorbance at 340 nm 10s after mixing, record as A1 measured/A1 standard, determine the absorbance at 340 nm 190 s after mixing, record as A2 measured/A2 standard, calculate ΔA = A2 − A1. Establish a standard curve, input ΔA to obtain *x* (μmol/mL) and ATP content (μmol/104 cell) = *x*/cell number.

### 2.10 Immunofluorescence Staining

The cells were cultured in copolymerised dishes (80% density), washed with PBS, fixed with 4% paraformaldehyde, permeabilised with 0.2%Triton X‐100, stained with Mito Tracker‐Blue, sealed, laser confocal microscope photographed and Adsorbed Secondary Antibody. Image J software was used for analysis.

The cells were cultured in copolymerised dishes (80% density), washed with PBS, fixed with 4% paraformaldehyde, permeabilised with 0.2%Triton X‐100, according to antibody: antibody diluent = 1: 1000 drops of diluted beta Actin Monoclonal Antibody (15G5A11/E2) (Thermo Fisher Scientific Inc., CN), incubated overnight at 4°C. Goat anti‐Mouse IgG (H + L) Cross‐Adsorbed Secondary Antibody, Alexa Fluor 488 (Thermo Fisher Scientific Inc., CN) dyed F‐actin, DAPI dyed nucleus, sealed, laser confocal microscope photographed and Adsorbed Secondary Antibody. Image J software was used for analysis.

TA muscle frozen sections were penetrated with 0.2% Triton X‐100 for 20 min at room temperature. Blocking was performed with 5% secondary antibody host serum for 40 min. After aspiration, the serum was washed three times with PBS for 5 min each time and incubated overnight at 4°C with the corresponding primary antibodies for detecting the target proteins (MYH7, MYH4 and MYH2). After removal of the primary antibody, the cells were washed three times with PBS for 5 min each time. The secondary antibody was incubated at room temperature for 1 h, and the secondary antibody was removed and washed three times with PBS for 5 min each time. The slides were sealed with anti‐fluorescence quenching agent (containing DAPI), and images were collected by laser confocal microscope. Image J software was used for analysis.

### 2.11 Flow Cytometry

ROS levels were determined using CellROX Green reagent (Thermo Fisher Scientific Inc., CN): 0.25% pancreatic enzyme digested C2C12 cells in a 6‐well plate, the digestion was terminated by double volume DMEM medium, EP tube was transferred, CellROX dye was added after PBS washing, PBS washing and suspension, flow tube was transferred and data were collected and analysed by flow cytometry.

### 2.12 Data Collection and Statistical Analysis

All data were represented by mean ± standard deviation. One‐way ANOVA and unmatched *t*‐test were used for statistical analysis for inter‐group comparison. GraphPad Prism 9 was used for mapping, and the significance levels were selected as *p < 0.05, **p < 0.01 and ***p < 0.001.

## 3 Results

### 3.1 Muscle Fibre Type Transformation After Skeletal Muscle Injury

**FIGURE 1:**
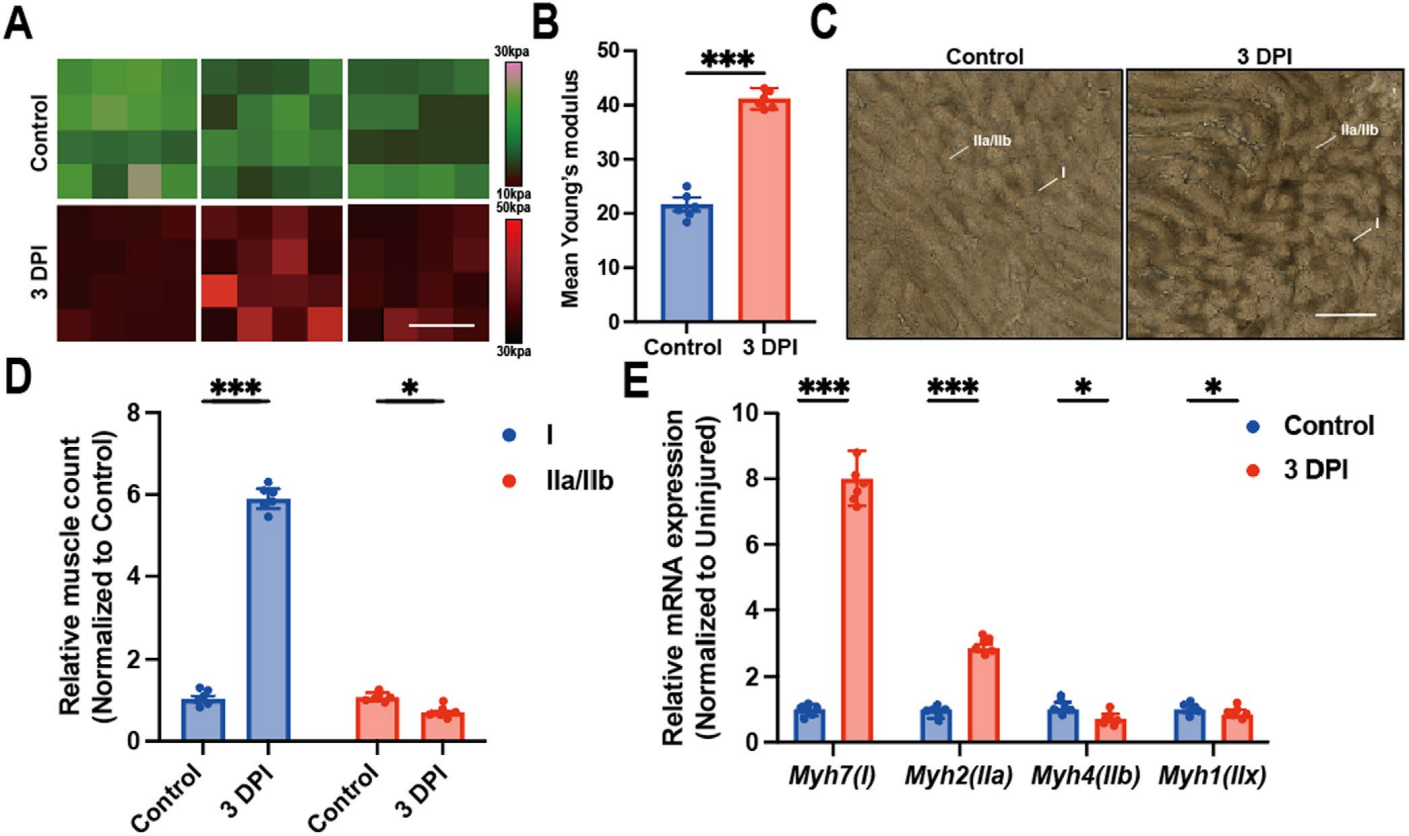
Matrix stiffness increases and muscle fibre types change after skeletal muscle injury. (A) Young's modulus before and after skeletal muscle injury was measured by atomic force microscopy (B), statistical analysis plot), scale 4um, ***p < 0.001, unpaired two tailed Student's *t*‐test. (C) Changes in muscle fibre type before and after skeletal muscle injury were detected by ATPase staining (D), statistical analysis plot), scale 100um, ***p < 0.001, *p < 0.05, two‐way ANOVA with Bonferroni's multiple comparisons. (E) Statistical map of mRNA relative expression levels of *Myh7, Myh2, Myh4* and *Myh1* after injury measured by timed fluorescent quantitative PCR, ***p < 0.001, *p < 0.05, two‐way ANOVA with Bonferroni's multiple comparisons.

Skeletal muscle stem cells can regulate skeletal muscle regeneration capacity by sensing mechanical and structural signals from the surrounding microenvironment [20]. To investigate the potential contribution of matrix stiffness changes to muscle fibre regeneration types after skeletal muscle injury, we first determined whether matrix stiffness changes occur after muscle injury. The Young's modulus of TA muscle was measured using atomic force microscopy (Figure 1A). The matrix stiffness was significantly increased 3 days after frostbite compared with control TA muscle (Figure 1B). Next, we used Atpase staining to detect changes in myofibre types in TA muscles (Figure 1C). As we mentioned in Methods, type I muscle fibres stained light brown and type IIa/IIb muscle fibres stained dark brown. The results showed that the number of type I muscle fibres in frostbite TA muscle increased significantly, and the number of type IIa/IIb muscle fibres decreased but without statistical difference (Figure 1D). Meanwhile, gene expression analysis by real‐time fluorescence quantitative PCR confirmed that the mRNA expression levels of *Myh7* and *Myh2* were significantly increased, while the mRNA expression levels of *Myh1* and *Myh4* were decreased in frostbite TA muscle (Figure 1E). These observations suggest that muscle fibre type transformation is affected by up‐regulation of matrix stiffness after skeletal muscle injury.

### 3.2 Increased Matrix Stiffness‐Induced Differentiation of C2C12 Cells Into Slow Muscle Fibres

**FIGURE 2:**
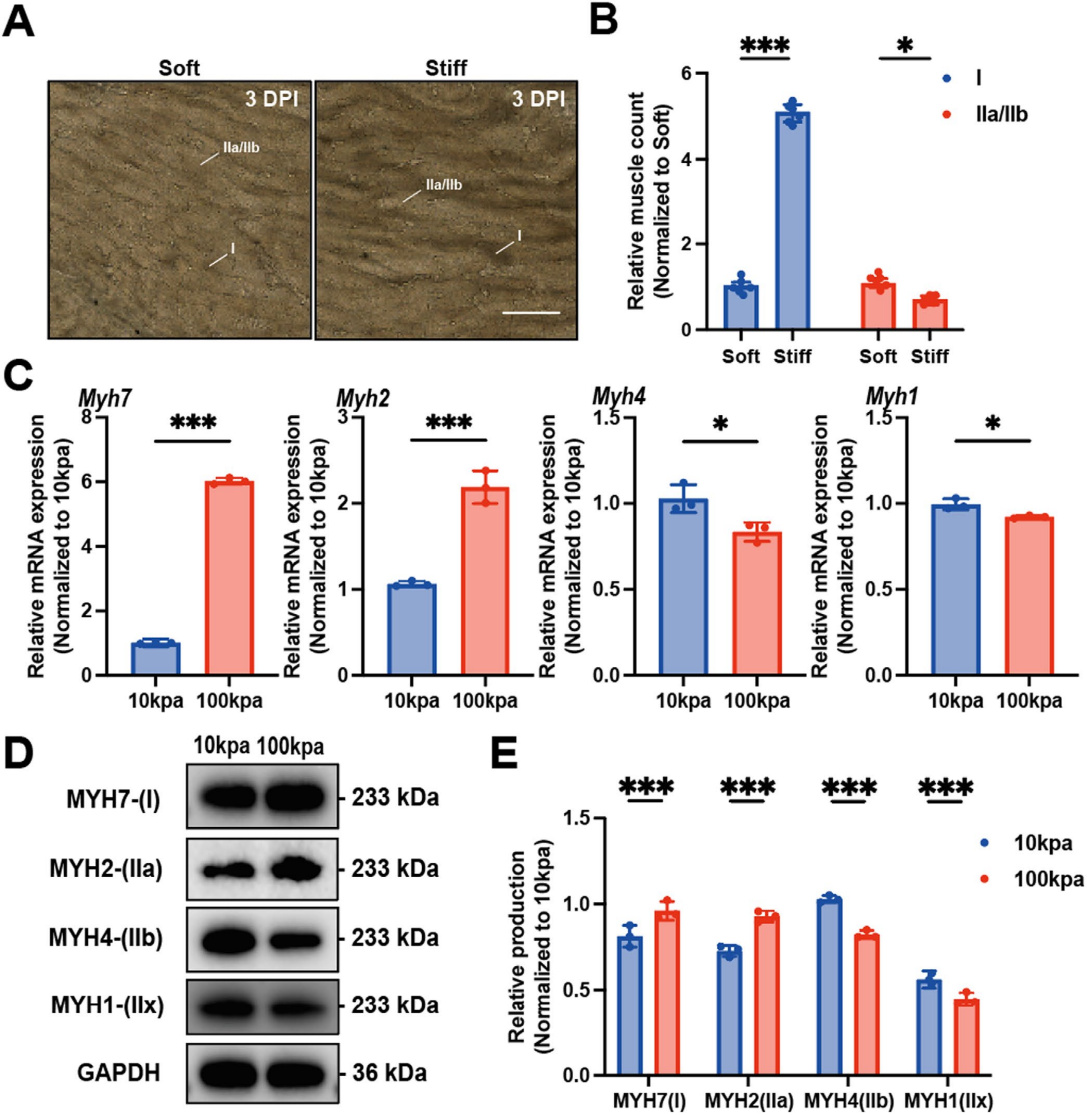
It was verified that matrix stiffness‐induced subtype changes of skeletal muscle stem cells. (A) Changes of muscle fibre types in different stiffness muscles were detected by ATPase staining (B, statistical analysis plot), scale 100um, ***p < 0.001, *p < 0.05, two‐way ANOVA with Bonferroni's multiple comparisons. (C) Statistical map of mRNA relative expression levels of *Myh7*, *Myh2*, *Myh4* and *Myh1* in C2C12 cells under different matrix stiffness measured by timed fluorescent quantitative PCR, ***p < 0.001, *p < 0.05, unpaired two tailed Student's *t*‐test. (D) Western blotting of MYH7, MYH2, MYH4 and MYH1 of C2C12 cells with different matrix stiffness (E, statistical analysis plot), ***p < 0.001, two‐way ANOVA with Bonferroni's multiple comparisons.

The influence of other physical and chemical factors on the transformation of muscle fibre types after skeletal muscle injury has not been excluded. TA muscles with different matrix stiffness ranges were selected for Atpase staining (Figure 2A). The average muscle stiffness less than 30 kpa was classified as soft TA muscle, and the average muscle stiffness more than 30 kpa was classified as hard TA muscle. It was found that compared with soft TA muscle, the number of type I muscle fibres in hard TA muscle showed an upward trend, and the number of type IIa/IIb muscle fibres showed a downward trend (Figure 2B), which proved that matrix stiffness played a major role in muscle fibre type transformation. Since the mechanical properties of ECM are almost impossible to control in vivo, we induced C2C12 cell differentiation in vitro using hydrogels with different stiffness to mimic the in vivo ECM. Gene expression analysis by real‐time fluorescence quantitative PCR confirmed that the mRNA expression levels of *Myh7* and *Myh4* in C2C12 cells cultured on 100 kpa hydrogel were significantly higher than those on 10 kpa hydrogel, while the mRNA expression levels of *Myh2* and *Myh1* were decreased (Figure 2C). Consistent with the mRNA expression level, the production levels of MYH7 and MYH4 were significantly increased, and the production levels of MYH2 and MYH1 were significantly decreased with the increase of matrix stiffness (Figure 2D,E) (Replicate data for the band plots of all experiments are available in Figure S3). These findings confirm that elevated matrix stiffness induces C2C12 cells to differentiate into slow myofibers.

### 3.3 Matrix Stiffness Changes the Energy Metabolism of Muscle Fibres

**FIGURE 3:**
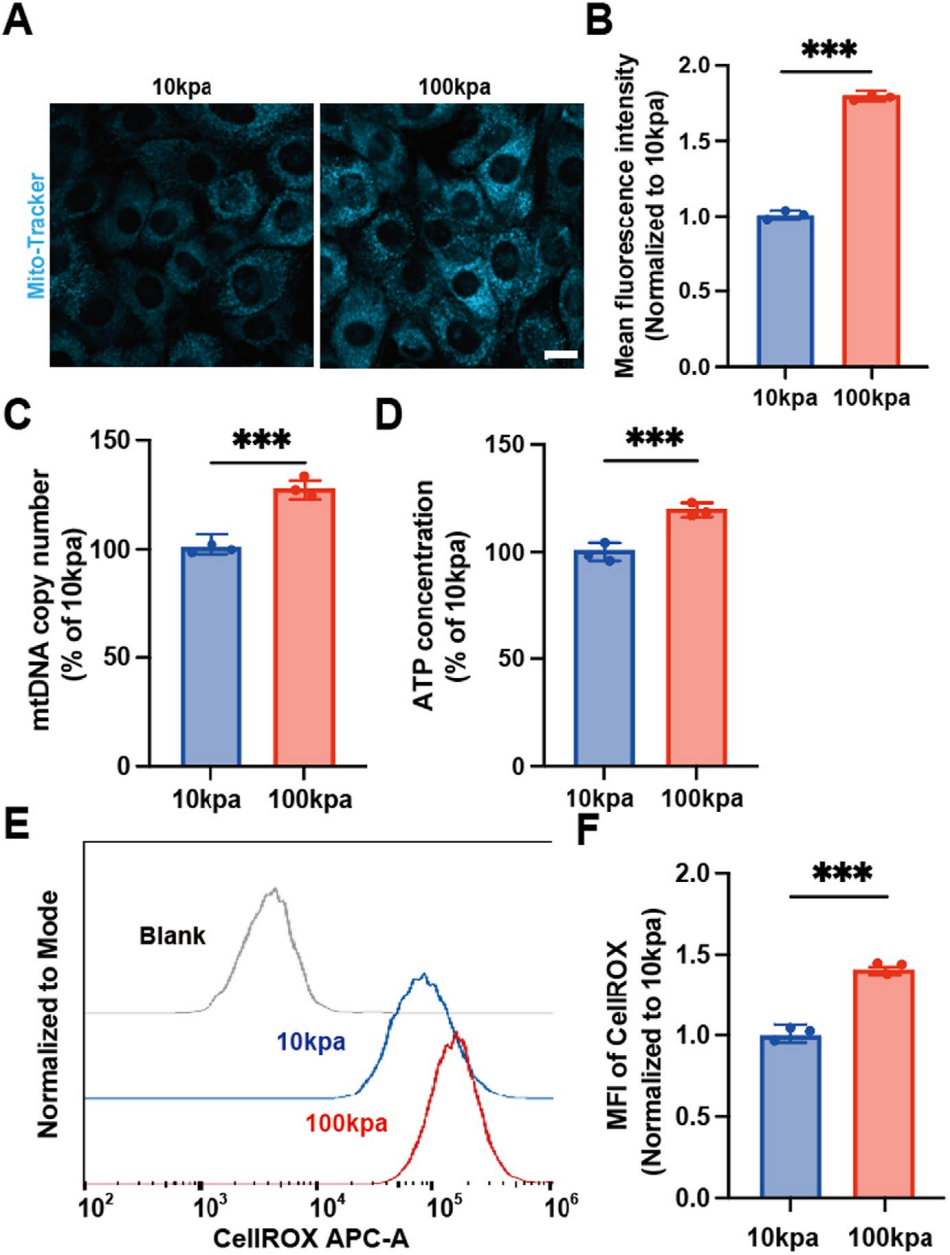
Matrix stiffness affects energy metabolism. (A) Mitochondrial Mito‐tracker fluorescence images of C2C12 cells with different matrix stiffness were captured by laser confocal microscope (B, statistical analysis plot), scale 10um, ***p < 0.001, unpaired two tailed Student's *t*‐test. (C) Relative statistical analysis of mitochondrial DNA copy number of C2C12 cells with different matrix stiffness, ***p < 0.001, unpaired two tailed Student's *t*‐test. (D) Relative statistical analysis of ATP production concentration in C2C12 cells with different matrix stiffness, ***p < 0.001, unpaired two tailed Student's *t*‐test. (E) CellROX curves of C2C12 cells with different matrix stiffness detected by flow cytometry (F, statistical analysis plot), ***p < 0.001, unpaired two tailed Student's *t*‐test.

Fast and slow muscle fibres are metabolised differently, we will further explore whether matrix stiffness affects the myogenic differentiation of C2C12 cells by regulating their energy metabolism. Mito‐Tracker Blue was used to stain mitochondria, and laser confocal imaging was used to detect the changes in the number of mitochondria in C2C12 cells under different matrix stiffness (Figure 3A). The results showed that the number of mitochondria was significantly increased at high matrix stiffness (Figure 3B). We reflected mtDNA copy number by *MT‐CO2* mRNA levels, and the results showed that mtDNA copy number was significantly increased at high matrix stiffness (Figure 3C). An increased number of mitochondria means more ATP synthase and the mitochondrial respiratory chain, which increases ATP production, while more electrons pass through the respiratory chain, increasing the electron leakage during oxidative phosphorylation, which in turn increases ROS production. In addition, increased mtDNA copy number can produce mtDNA‐encoded respiratory chain complex subunits, thereby enhancing ATP production [31]. Taken together, this prompted us to further examine mitochondrial function (ATP content and ROS levels). The results showed that ATP content was significantly increased (Figure 3D), and ROS level was significantly increased (Figure 3E,F). These findings all suggest that elevated matrix stiffness can increase the number of mitochondria in C2C12 cells and promote a shift in muscle fibre metabolism toward oxidative phosphorylation.

### 3.4 Matrix Stiffness Remodels the Cytoskeleton to Regulate Mitochondrial Division

**FIGURE 4:**
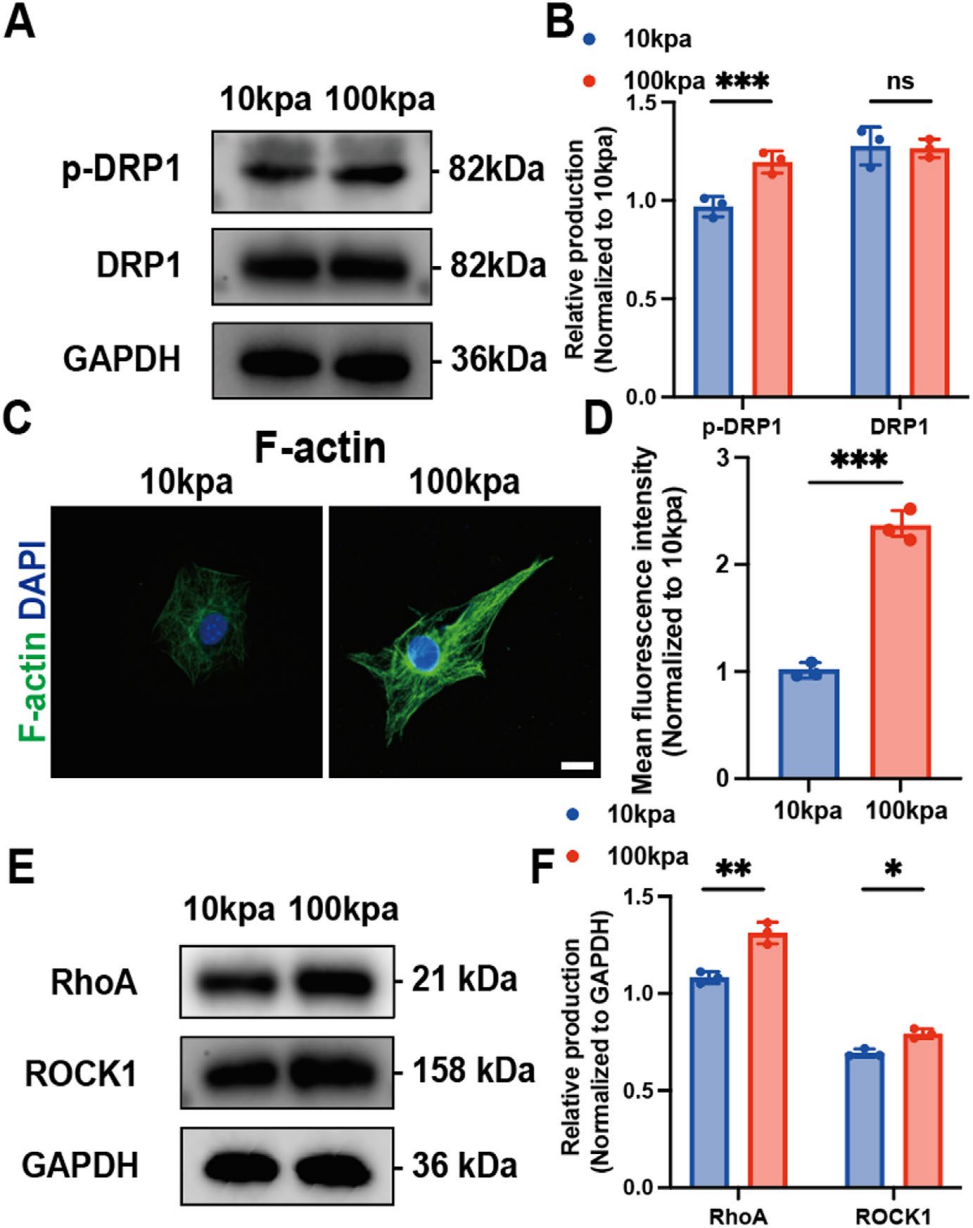
Matrix stiffness remodelling Actin activated DRP1. (A) Western blotting of p‐DRP1 and DRP1 in C2C12 cells with different matrix stiffness. (B, statistical analysis plot), ***p < 0.001,  ns, no significance, two‐way ANOVA with Bonferroni's multiple comparisons. (C) Immunofluorescence images of C2C12 cytoskeleton protein F‐actin with different matrix stiffness captured by laser confocal microscopy. (D, statistical analysis plot), ***p < 0.001, unpaired two tailed Student's *t*‐test. (E) Western blotting of RHOA, ROCK1 of C2C12 cells with different matrix stiffness (F, statistical analysis plot), **p < 0.01, *p < 0.05, two‐way ANOVA with Bonferroni's multiple comparisons.

Mitochondrial division is driven by dynamin‐related protein 1 (DRP1), which is recruited from the cytoplasm to the mitochondrial outer membrane mediated by actin filaments, oligomerisation into a contractile ring and splitting one mitochondrion into two daughter mitochondria [32, 33]. Notably, division in the middle of a mitochondrion leads to its proliferation. However, the division close to the periphery causes the damaged part to shed into smaller mitochondria, triggering mitophagy [34]. Therefore, we speculate that the increased number of mitochondria at high matrix stiffness is caused by mitochondrial fission. Western blot was used to detect the phosphorylation of DRP1 in cells with different matrix stiffness, and it was observed that the level of p‐DRP1 in the 100 kpa group was significantly increased (Figure 4A,B). This suggests that DRP1 phosphorylation can promote mitochondrial fission and increase mitochondrial number. The above mentioned recruitment of DRP1 by actin filaments to mitochondrial outer membrane receptors prompted us to further explore whether the actin cytoskeleton isffected by matrix stiffness. The results of immunofluorescence staining showed that the mean fluorescence intensity of F‐actin in C2C12 cells was significantly increased under high matrix stiffness (Figure 4C,D), suggesting that matrix stiffness was involved in cytoskeleton remodelling. It has been reported that increased matrix stiffness in tumours increases Rho/ROCK‐dependent cytoskeletal tension [35, 36, 37]. Western blot results showed that high matrix stiffness up‐regulated the production of RHOA and ROCK1 in C2C12 cells (Figure 4E,F), and activated the RHO/ROCK pathway. Taken together, matrix stiffness transmits mechanical signals that activate the RHO/ROCK pathway, reshape the actin skeleton and activate DRP1 translocation to mitochondria, leading to contractile ring assembly and mitochondrial outer membrane contraction.

### 3.5 Targeting Cytoskeleton to Regulate Myogenic Differentiation of C2C12 Cells

**FIGURE 5:**
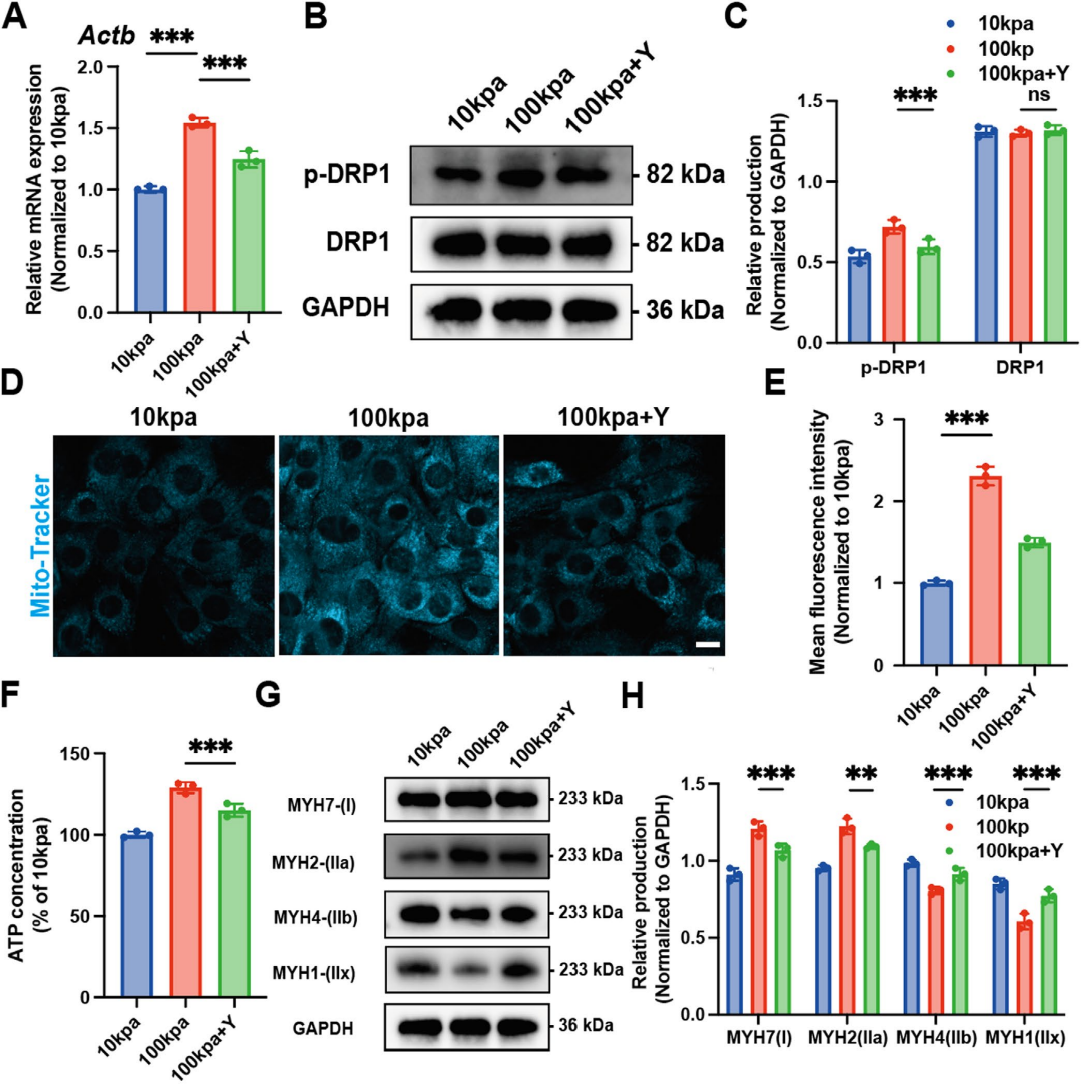
Targeting Actin regulates skeletal muscle stem cell differentiation phenotype. (A) Relative statistical analysis of mRNA of Actb of C2C12 cells with different matrix stiffness detected by timed fluorescent quantitative PCR using Y‐27632, ***p < 0.001, two‐way ANOVA with Bonferroni's multiple comparisons. (B) Western blotting of p‐DRP1 and DRP1 of C2C12 cells with different matrix stiffness using Y‐27632. (C, statistical analysis plot) ***p < 0.001, two‐way ANOVA with Bonferroni's multiple comparisons. (D) Confocal laser microscopy using Y‐27632 mitochondrial Mito‐tracker staining fluorescence images of C2C12 cells with different matrix stiffness (E, statistical analysis plot), scale 10um, ***p < 0.001, two‐way ANOVA with Bonferroni's multiple comparisons. (F) Relative statistical analysis of ATP production concentration in C2C12 cells with different matrix stiffness using Y‐27632, ***p < 0.001, unpaired two tailed Student's *t*‐test. (G) Western blotting of C2C12 cells MYH7, MYH2, MYH4 and MYH1 using Y‐27632 with different matrix stiffness (H, statistical analysis plot), ***p < 0.001, **p < 0.01, two‐way ANOVA with Bonferroni's multiple comparisons.

The ROCK inhibitor Y‐27632 was added to the 100 kpa group for pretreatment for 1 day. RNA was extracted from C2C12 cells in the 10 kpa group, the 100 kpa group and the 100 kpa group pretreated with Y‐27632 for 1 day, respectively. After reverse transcription, real‐time fluorescence quantitative PCR was performed. The results showed that the mRNA expression level of *Actb* in C2C12 cells was significantly increased under high matrix stiffness (Figure 5A). However, this change was reversed by the addition of Y‐27632. Notably, the addition of Y‐27632 did not completely reverse the up‐regulation of Actb transcript levels by high matrix stiffness, indicating the existence of signalling pathways other than RHO/ROCK involved in the regulation of the actin skeleton. We also found that the p‐DRP1 level was downregulated by Y‐27632 (Figure 5B,C), again demonstrating that the actin cytoskeleton mediates the activation and translocation of DRP1. So far, our data suggest that Y‐27632 prevents the RHO/ROCK pathway from inducing cytoskeletal remodelling and inhibiting mitochondrial fission. To further support this idea, we observed that Y‐27632 significantly reduced mitochondrial number (Figure 5D,E). At the same time, the significant decrease of ATP content indicated that mitochondrial function was inhibited (Figure 5F). Moreover, addition of Y‐27632 significantly reversed the high matrix stiffness‐induced up‐regulation of MYH7 and MYH2 and down‐regulation of MYH4 and MYH1 (Figure 5G,H). Intraperitoneal injection of Y‐27632 in mice and in vivo detection of changes in muscle fibre types showed a consistent trend with the in vitro experiments. Y‐27632 significantly reversed the up‐regulation of Myh7 and the down‐regulation of Myh4 and Myh1 induced by frostbite (Figures S1, S2). Taken together, these results indicate that matrix stiffness induces cytoskeletal remodelling, promotes mitochondrial fission and promotes C2C12 cells differentiation into slow muscle fibres.

## 4 Discussion

In the study by Silver et al. [19] matrix stiffness decreased two‐fold 5 days after TA muscle injury and then continued to increase while in the present study, matrix stiffness increased 3 days after TA muscle injury, and the difference between our results and their results may be attributed to differences in injury mode. The discrepancy between our results and theirs may be attributed to differences in the mode of injury. We used a frostbite model, Silver et al. used a chemical (BaCl2) injury model, and elsewhere, mechanical injuries such as contusion [38] and crush [39], chemical injuries such as injection of cardiotoxin (CTX) and Notexin (NTX) [40], and hypoxic–ischemic and ischemia–reperfusion injuries. It should be emphasised that there may be some differences in the changes caused by different damage modes. This study only focuses on frostbite damage and does not discuss and verify other damage modes too much. The ECM transmits mechanical signals to cells through the collagen, integrin and actin cytoskeleton, and regulates the proliferation and differentiation of SCs [5, 21, 41]. We hypothesised that elevated matrix stiffness caused by collagen deposition would affect the direction of myogenic differentiation of SCs. Results showed that high matrix stiffness increased the expression of slow fibre/intermediate fibre signature genes (*Myh7/Myh2*), while decreased the expression of fast fibre signature genes (*Myh4* and *Myh1*), which promoted the conversion of fast fibre into slow fibre. Silver et al. and Madl et al. [19, 42] have used hydrogels to simulate ECM hardening and found that the proliferation and migration ability of SCs increased with the increase of matrix stiffness, but stiff hydrogels impaired the proliferation ability of SCs and prevented myogenic progression. In this study, we inoculated C2C12 cells with 10 kpa and 100 kpa hydrogels, respectively, and found that the mRNA expression levels of *Myh7* and *Myh2* were highly up‐regulated in cells seeded with high matrix stiffness, suggesting myogenic differentiation of C2C12 cells toward slow muscle fibres.

More and more studies have proved that most of the differentiated SCs turn to oxidative phosphorylation, which is essential for terminal myogenic differentiation [5]. At the same time, reduced mitochondrial network remodelling has been repeatedly shown to reduce the differentiation ability of cultured myoblasts and reduce the regeneration ability of skeletal muscle tissue [43]. In the study by Hong et al. [44] they selected SCs from the regenerated muscle at 3 days after injury (3DPI‐SCs) and found that mitochondrial fission related genes (*Drp1, Fis1* and *Mff*) were significantly up‐regulated in 3DPI‐SCs compared to the uninjured muscle SCs, and the same results were obtained at the protein level. This eventually leads to an increase in the number of mitochondria. Similar findings were found in the present study. Through immunofluorescence staining and transcriptome analysis, we found that compared with the 10 kpa group, the number of mitochondria and mtDNA copy number of C2C12 cells in the 100 kpa group were significantly increased, suggesting that mitochondrial function was enhanced. The ATP absorbance was detected by ultraviolet spectrophotometer, and the fluorescence intensity of ROS staining was detected by flow cytometry. ATP production and ROS scavenging ability were found to be enhanced. These results suggest that myoblasts increase the number of mitochondria and enhance mitochondrial function to meet the metabolic demand of muscle fibres during differentiation.

In order to adapt to the changing energy demand of muscle fibres, cells dynamically reconstitute mitochondrial dynamics (division and fusion). When the division rate is greater than the fusion rate, the number of mitochondria is increased. As mentioned above, mitochondrial division requires DRP1 drive, which is a Gtpase. DRP1 is regulated by a variety of post‐translational modifications, including phosphorylation and SUMOylation. DRP1 can activate and inhibit according to the phosphorylation modification of specific sites [45]. Ser616 and Ser637 (corresponding to Ser579 and Ser600 in mice, respectively) are two major sites involved in the regulation of mitochondrial fission activity [46]. Phosphorylation at Ser‐616 facilitates DRP1 localisation to the mitochondrial outer membrane and subsequent contractile division [45]. Interestingly, Brand et al. [47] found that activation of the RHOA/ROCK pathway in mouse cardiomyocytes phosphorylates DRP1 at Ser616 and mediates DRP1 localisation to mitochondria, thereby transferring environmental signals to mitochondria. The role of phosphorylation at Ser637 remains highly controversial. Although most reports suggest that phosphorylation at Ser637 is associated with decreased gtpase hydrolytic activity of DRP1, its phosphorylation is different in different environments. For example, Juhn et al. [48] found that PKD causes mitochondrial fragmentation and dysfunction in cardiomyocytes by phosphorylating Ser637. Similarly, Wang et al. [49] found that the ROCK1‐mediated metabolic pathway in podocytes involved the phosphorylation of DRP1 at Ser637, promoting its translocation to mitochondria and causing mitochondrial fission. Since the specific phosphorylation site of DRP1 was not involved in this study, it will not be discussed here. In the present study, the p‐DRP1 level was found to be increased in cells with high matrix stiffness, a result consistent with our expectation. In addition, the actin skeleton recruits DRP1 from the cytoplasm to mitochondria, allowing them to oligomerise further to form contractile rings. We examined the changes of actin skeleton in cells with different matrix stiffness, and found that the fluorescence intensity of F‐actin protein skeleton was significantly increased in the 100 kpa group. We also observed that F‐actin protein skeleton was significantly aggregated in the perinuclear region, which may be related to mitochondrial migration.

In this study, thin‐layer coating method was used to culture C2C12 cells, and the cells can sense the stiffness of the hydrogel by applying contractile force through stress fibres attached to the hydrogel, thereby activating various proteins at the adhesion site, such as adhesion kinase, RHO and ROCK [50]. Upon binding of activated RHOA to ROCK, it causes cytoskeletal remodelling [51]. We observed that high matrix stiffness induced the production of RhoA and ROCK1 proteins in the RHO/ROCK pathway and activated the RHOA/ROCK1 pathway. Patyal et al. found that inhibition of RhoA downregulated mitochondrial gene expression levels and reduced ATP production. It also inhibits SRF and reduces the formation of F‐actin [52]. In our study, pretreatment with the ROCK1 inhibitor Y‐27632 to the differentiation medium for 1 h resulted in a significant down‐regulation of Actb mRNA expression levels in cells, demonstrating that RhoA/ROCK1 pathway activation can remodel the actin skeleton. In addition, RHOA/ROCK1 in cardiomyocytes [47] and ROCK1 in podocytes [43] phosphorylate DRP1 at different sites, respectively, and mediate DRP1 targeting to the mitochondrial surface. Although different cell types, we observed a similar result that inhibition of ROCK1 downregulated the phosphorylation level of DRP1. Taken together, the RHO/ROCK pathway can mediate the translocation of actin to mitochondria either by remodelling the actin skeleton or by directly activating DRP1. Addition of Y‐27632 also reversed the increase in mitochondrial number and the up‐regulation of MYH7 and MYH2 protein production levels at high matrix stiffness, revealing the important role of RHO/ROCK pathway in matrix stiffness affecting myofiber differentiation.

In conclusion, our study investigated the role of matrix stiffness in myofibre type transformation during skeletal muscle injury repair and identified the underlying regulatory mechanisms. We found that skeletal muscle injury was accompanied by elevated ECM stiffness. More importantly, we found that SCs induced mitochondrial fission by sensing ECM stiffness changes, activating the RHO/ROCK pathway, remodelling the actin cytoskeleton and phosphorylating DRP1, and enhancing oxidative metabolic capacity to promote SCs differentiation into slow muscle fibres. These results reveal that after skeletal muscle injury, the increase of matrix stiffness can promote the conversion of fast muscle fibres to slow muscle fibres during the repair process of injury, and provide a new theoretical basis and therapeutic strategy for the treatment of muscle diseases.

## Author Contributions

**Dongmei Wang:** data curation (equal), funding acquisition (equal), writing – original draft (equal). **Jiahong Wu:** data curation (equal), writing – original draft (equal). **Zeyu Xu:** data curation (equal), writing – original draft (equal), writing – review and editing (equal). **Jinning Jia:** writing – original draft (equal), writing – review and editing (equal). **Yimei Lai:** data curation (equal), writing – original draft (equal). **Zhihua He:** resources (equal), supervision (equal), writing – review and editing (equal).

## Ethics Statement

This study was approved by the Experimental Animal Management and Use Committee of Shenzhen TopBiotech Co. Ltd. (TOPGM‐IACUC‐2024‐0057). We confirm that we have read the Journal's position on issues involved in ethical publication and affirm that this report is consistent with those guidelines.

## Conflicts of Interest

The authors declare no conflicts of interest.

## Supporting information


Figures S1–S3.

## Data Availability

The data supporting the findings of this study can be obtained from the corresponding author upon reasonable request.
