# Supplementary material for: Increased Matrix Stiffness Promotes Slow Muscle Fibre Regeneration After Skeletal Muscle Injury
Source: J Cell Mol Med. 2025 Feb 19;29(4):e70423. doi: 10.1111/jcmm.70423 (PMC11837045; doi:10.1111/jcmm.70423)
Supplement: Supplementary file 1 — Figures S1–S3. [file JCMM-29-e70423-s001.pdf]

SFig.1

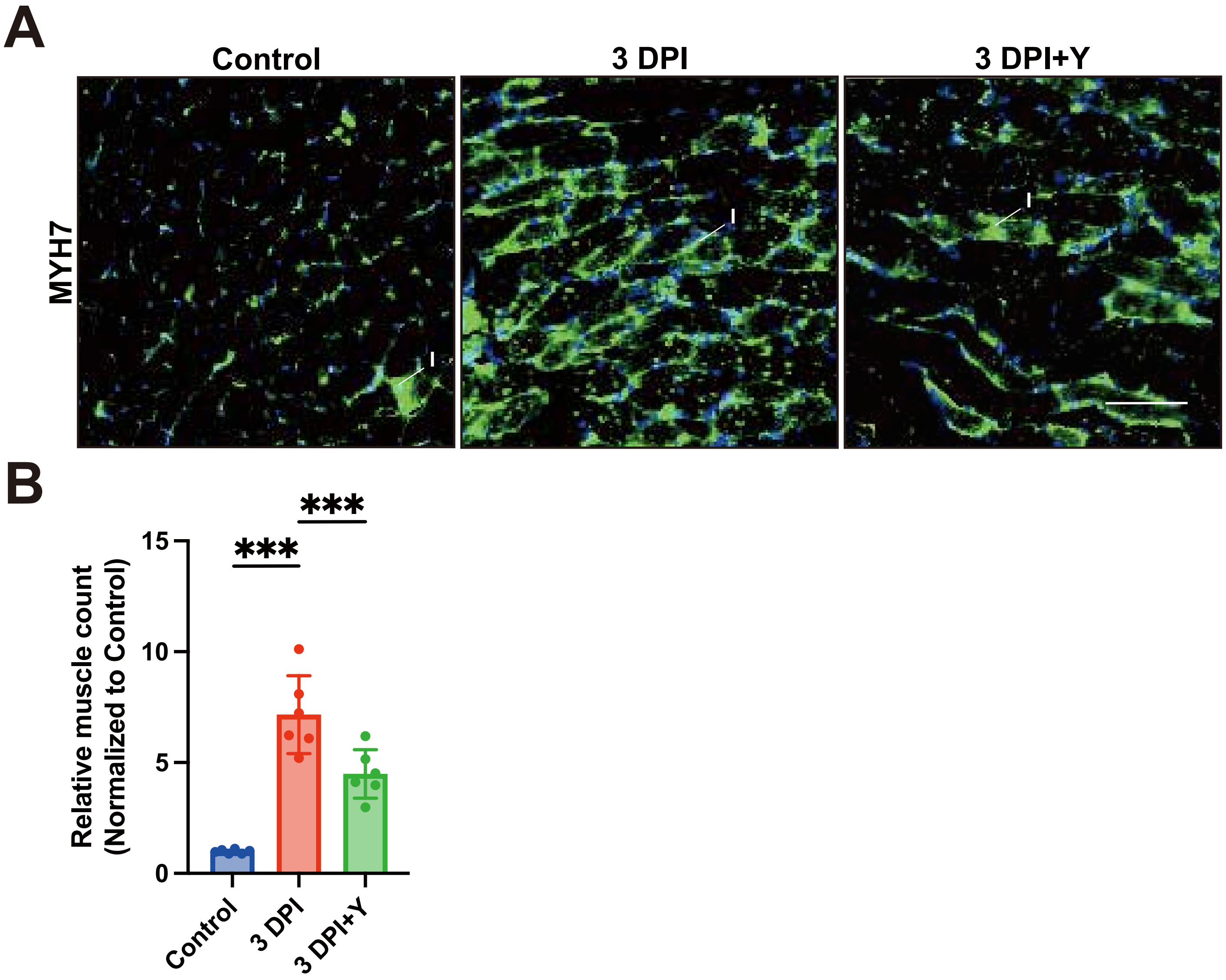

(A) Immunofluorescence images of MYH7 before and after TA injury using Y-27632 captured by laser confocal microscopy. (B, statistical analysis plot), ***P < 0.001, two-way ANOVA with Bonferroni's multiple comparisons.

SFig.2

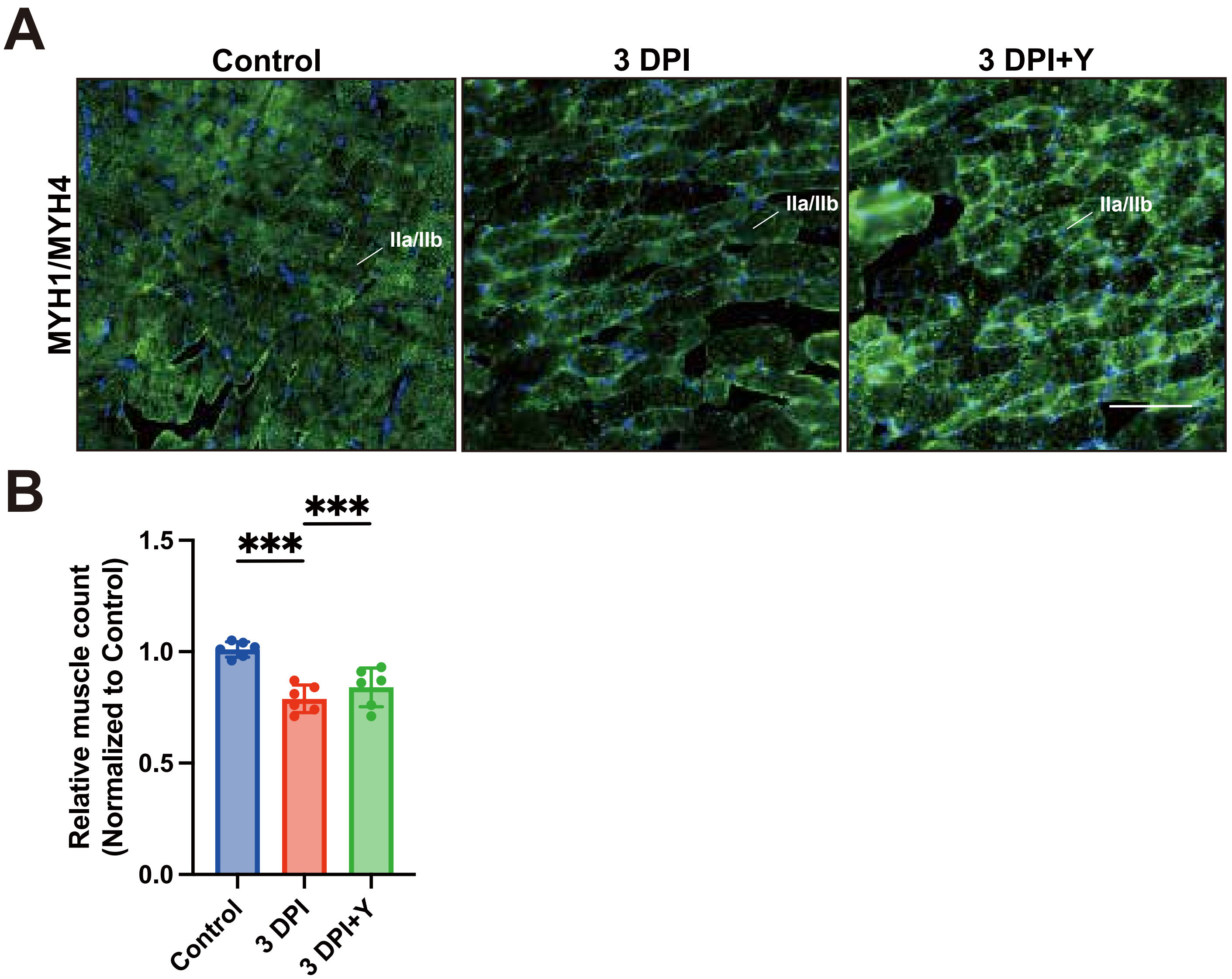

(A) Immunofluorescence images of MYH1/MYH4 before and after TA injury using Y-27632 captured by laser confocal microscopy. (B, statistical analysis plot), ***P < 0.001, two-way ANOVA with Bonferroni's multiple comparisons.

SFig.3

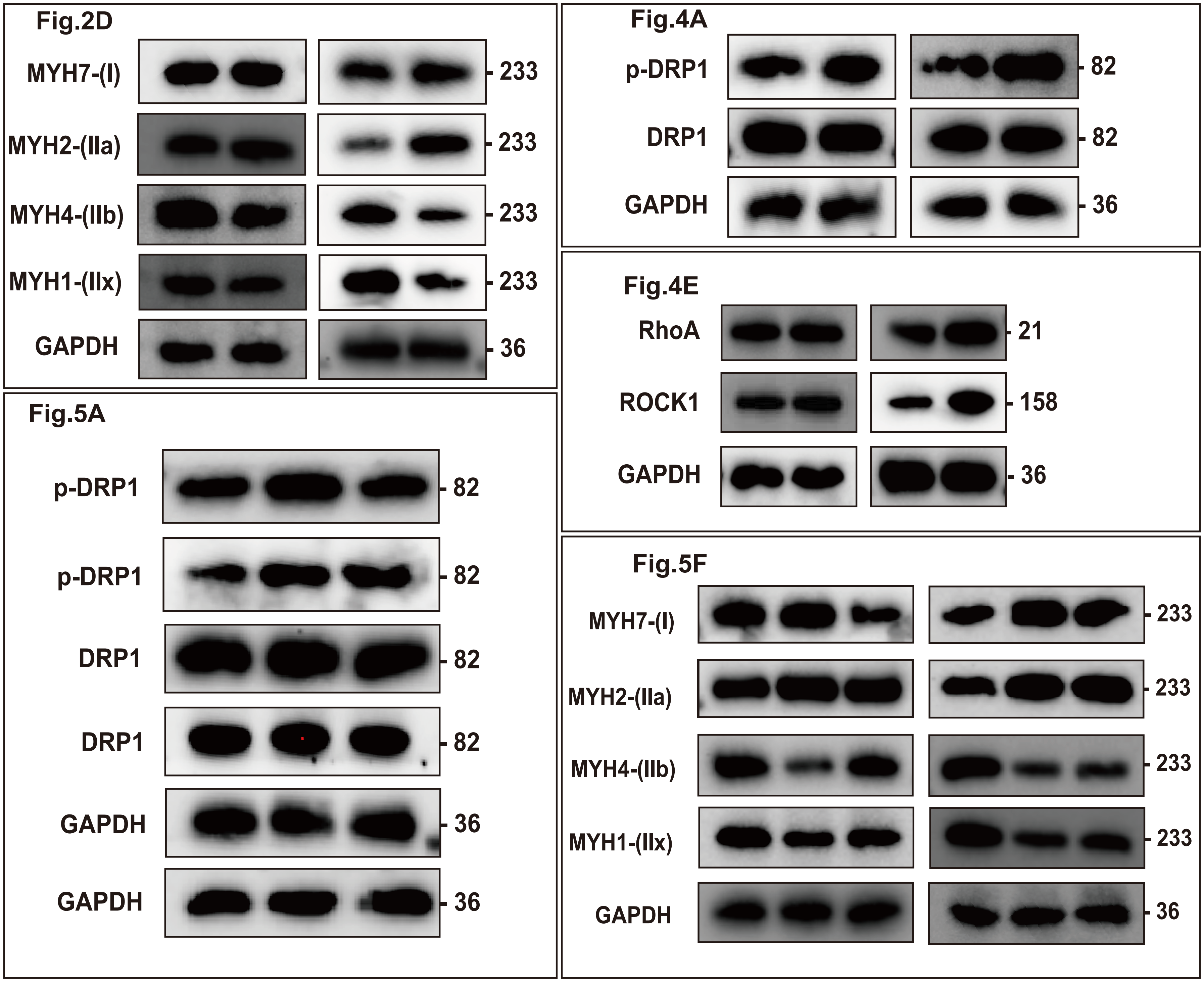

Duplicate data from WB images
